# An Entropy-Based Combined Behavior Model for Crowd Evacuation

**DOI:** 10.3390/e24101479

**Published:** 2022-10-17

**Authors:** Xiaowei Chen, Jian Wang

**Affiliations:** Department of Electronic and Information Engineering, Tongji University, Shanghai 201804, China

**Keywords:** crowd simulation, entropy, evacuation behavior, small social groups

## Abstract

Crowd evacuation has gained increasing attention due to its importance in the day-to-day management of public areas. During an emergency evacuation, there are a variety of factors that need to be considered when designing a practical evacuation model. For example, relatives tend to move together or look for each other. These behaviors undoubtedly aggravate the chaos degree of evacuating crowds and make evacuations hard to model. In this paper, we propose an entropy-based combined behavior model to better analyze the influence of these behaviors on the evacuation process. Specifically, we utilize the Boltzmann entropy to quantitatively denote the degree of chaos in the crowd. The evacuation behavior of heterogeneous people is simulated through a series of behavior rules. Moreover, we devise a velocity adjustment method to ensure the evacuees follow a more orderly direction. Extensive simulation results demonstrate the effectiveness of the proposed evacuation model and provide useful insights into the design of practical evacuation strategies.

## 1. Introduction

Crowd evacuation in congested indoor scenes (e.g., due to emergencies) is an important issue, which should be considered in building designs and the day-to-day management of public areas. Because it is difficult to conduct practical exercises and there is a lack of data on crowd populations, computer simulation is widely adopted in the study of crowd evacuation problems.

Evacuation models based on computer simulations typically include two types: macro evacuation, which considers the population as a homogeneous flow [1,2], and micro evacuation, which analyzes the behavior characteristics of individuals during crowd evacuation. In particular, micro evacuation models have gained more attention due to fact that they can better investigate the crowd’s evolution trend. Classic micro evacuation models include the social force models [3], cellular automaton models [4], agent simulation models [5], and hybrid models [6,7]. These models are utilized for optimizing escape strategies and routes, improving guidance information, identifying building bottlenecks, and predicting evacuation times [8,9,10,11,12,13,14].

However, the existing works have two main limitations. First, these works do not consider the impact of crowd disorder on the evacuation process, which is an implicit but important indicator in many real-world evacuation scenarios. For example, if the crowd is more chaotic, the evacuees would be more likely to behave irrationally, leading to a longer evacuation time or even a mass stampede. Therefore, the degree of crowd disorder can affect individual decision making. Second, these works often focus on a single evacuation behavior, lacking a comprehensive mechanism to describe the various behaviors of heterogeneous crowds, which is why it is difficult to model an evacuation in practice. For example, there are various factors that affect an evacuation simultaneously, such as environmental familiarity, the social relationships of individuals among small social groups, and individuals in heterogeneous populations, making the dynamics of crowd evacuation more complex, which in turn, also influences crowd disorder.

To address these limitations, in this paper, we propose an Entropy-based Combined Behavior Model (ECBM), which incorporates the degree of crowd chaos into an evacuation. In particular, we adopt the Boltzmann entropy to quantify the degree of crowd chaos and utilize the chaos degree to adjust the velocities of a variety of evacuation behaviors. This novel design allows us to better capture the behaviors of individuals in the evacuation process, making it more realistic and accurate. Furthermore, we investigate the influence of kinship relationships and guides by carefully analyzing their effects on different evacuation behaviors and integrating them into the evacuation model. Specifically, we make the following contributions:

- We propose an entropy-based combined behavior model ECBM that combines a variety of evacuation behaviors, making crowd evacuation more realistic.
- We integrate the crowd chaos based on the Boltzmann entropy into the ECBM and present a velocity adjustment method to ensure the evacuees follow a more orderly direction.
- We implement the ECBM and conduct extensive experiments using simulations to evaluate the ECBM. The simulation results validate the effectiveness of our evacuation model.

The rest of this paper is organized as follows. Section 2 introduces the related work. In Section 3, we introduce the detailed design of the ECBM. We demonstrate the effectiveness of the ECBM through simulations in Section 4. Finally, we conclude the paper in Section 5.

## 2. Related Work

During an evacuation process, individuals have some typical behaviors. For example, Konstantara et al. [15] classified an evacuation crowd according to the moving speed of pedestrians and proposed a cellular automaton model to study the role of professionals in assisting the elderly with weak mobility during an evacuation. Considering the disability of pedestrians, Mitsopoulou et al. [16] divided the evacuation crowd into six categories, established an agent-based model to simulate the evacuation process of heterogeneous crowds, and analyzed the impact of the physical and psychological attributes of disabled people on crowd evacuation. In particular, small social groups have gained increasing attention in relation to crowd evacuation. There are significant differences in the micro-behavioral characteristics of small social groups and individuals [17,18]. Behavioral interactions within social groups can affect the overall evacuation dynamics of the population [19]. Although some models consider the behavior of slowing down and waiting for lagging members in small groups [20], most studies only focus on simulating the gathering behavior of small social groups during an evacuation by introducing the leader–follower mechanism or group attraction force [21]. Aiming at the kinship-related small social groups that commonly exist in evacuating populations, Yang et al. [22] described the behavioral interactions within small groups, such as gathering and backtracking, through kinship attraction. However, in their model, members of the same group are globally visible without considering the limitations of the individual’s field of vision. In addition, for evacuees familiar with the building environment and exit locations, the moving target is often clear and they can also guide others to evacuate through information sharing. In most crowd evacuation models, the behavior of such evacuees is often reflected in the guiding behavior [23]. Some evacuation models simulate the behavioral interactions between guides and non-guides by introducing a guiding attraction force [24,25]. However, these models only consider one specific behavior, lacking a comprehensive description of the various behaviors of heterogeneous evacuation crowds; thus, these models are not realistic enough to simulate crowd evacuation.

For the heterogeneous evacuation crowd composed of small social groups, guides, and individuals, the diversity and complexity of behavior also affect the crowd’s chaos degree and evacuation efficiency. Some studies showed that people with stable relationships (e.g., friends or lovers) have obvious characteristics in an emergency evacuation [26]. In addition, some existing works found that the evacuation time increases if each group size is small (e.g., two or three) [27,28,29], whereas the time decreases when the group size further increases (e.g., larger than three) [30,31]. The research shows that the presence of guides also has a dual effect on crowd evacuation and is closely related to visibility and local crowd density [24,25,32,33]. Nevertheless, these studies only investigated the evacuation efficiency from a single perspective, without considering the comprehensive effect of these small social groups and guides on the evacuation time and crowd disorder.

The complex evacuation behavior of heterogeneous crowds significantly impacts the crowd evacuation dynamics. At the same time, local crowd chaos also interferes with the behavior in the movement of individuals. Entropy is one of the most common methods used for measuring crowd disorder in an evacuation [34,35,36,37]. For instance, Rangel-Huerta et al. [35] utilized entropy to denote the heterogeneity of evacuees, Zhao et al. [36] used Shannon entropy to evaluate population mutations, and Huang et al. [37] adopted the attenuation of velocity entropy to identify congestion areas. However, considering the limited perceptual range of evacuees, it is almost impossible for evacuees to accurately calculate the dynamics of the global crowd disorder. Thus, in the ECBM, we utilize local disorder to reflect the influence of crowd disorder on evacuation individuals.

## 3. ECBM Model

**Figure 1:**
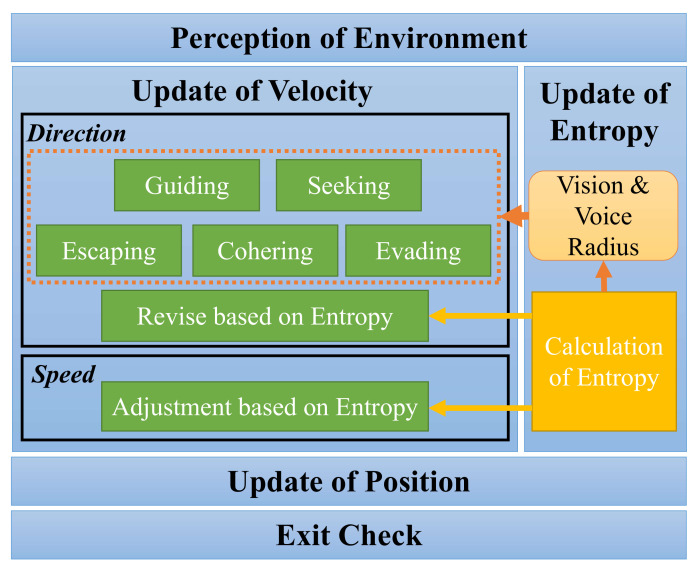
The ECBM framework.

In this section, we present our entropy-based combined behavior model (ECBM), which incorporates the indicator of crowd chaos into the evacuation process. The ECBM framework is illustrated in Figure 1. We consider the multi-agent simulation method, where an individual (i.e., agent) is represented as a circular particle with a set of attributes (e.g., velocity, visual field). To make the model more realistic, we consider three types of agents: (1) individuals who make the decision independently; (2) individuals who have pairwise kinship relationships, i.e., small social groups; and (3) individuals who are familiar with the structure of the scene, that is, they can act as guides to lead the evacuation. We further design five modules to describe the various behaviors of agents based on the agent type and the Boids rules [38], which we detail in Section 3.1. Given the agent behaviors, in Section 3.2, we present the detailed ECBM algorithm, including the Boltzmann entropy calculation that quantifies the chaotic level of the visible crowd from the perspective of each agent as well as the velocity adjustment of individuals so that the simulated agents can evacuate in a more orderly direction.

### 3.1. Behavior Modules

For each step of an evacuation, we need to calculate the velocity (i.e., moving speed and moving direction) of each agent toward the exit. In particular, we consider five behaviors in the ECBM, including the three basic behaviors (escaping, evading, and cohering) for all the agents, the seeking behavior for agents in small social groups, and the guiding behavior for guides. In the following subsections, we present the design of each behavior, respectively.

**Figure 2:**
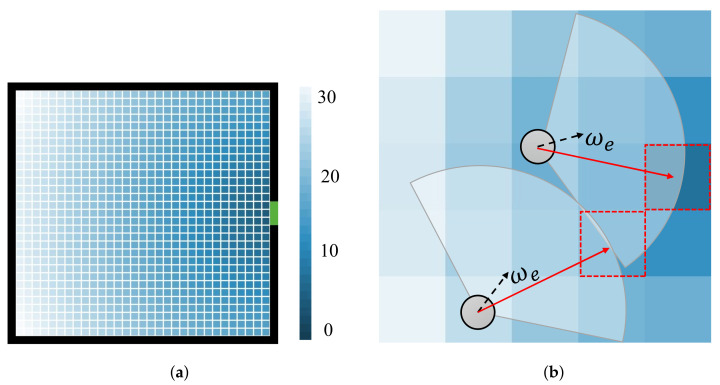
Illustration of escaping behavior. (**a**) Escape floor field; (**b**) Escaping behavior.

Escaping behavior. The escaping behavior refers to the behavior of an individual moving toward the exit. During an indoor evacuation, humans usually rely on their memory of the exits to distinguish the desirable direction in which to escape. The escaping of agents in the ECBM is based on an escape floor field, similar to the static field in the floor field model. The evacuation scene is discretized into a one-meter-square grid. The value of the escape floor field for individuals is equal to the distance from each square to the center of the exit, as in Figure 2a.

Figure 2b gives an illustration of this behavior. It can be seen that some studies only focus on the field of view radius and do not limit the field of view angle, which is set to 360° [39]. However, during an evacuation, pedestrians usually pay more attention to the front environment than the rear or sides. Therefore, several models set the field of view angle to 180° [40] or 170° [41]. Considering that panic may lead to the narrowing of the pedestrian’s field of vision [42], we assume that the visual field of the agent is a sector with an angle of 160° and a radius of $d_{\mathrm{vision}}$. Agents prefer to choose the lowest escape floor field in the visible area as the escape direction. The steering angle $w_{e}$ in Figure 2b suggests that individuals deflect as much as possible to the optimal goal. In the ECBM, we set the extreme value for this parameter $\Phi_{e}$. This threshold reflects the attraction of the exit to individuals or the individual’s ability to remember the exits.

**Figure 3:**
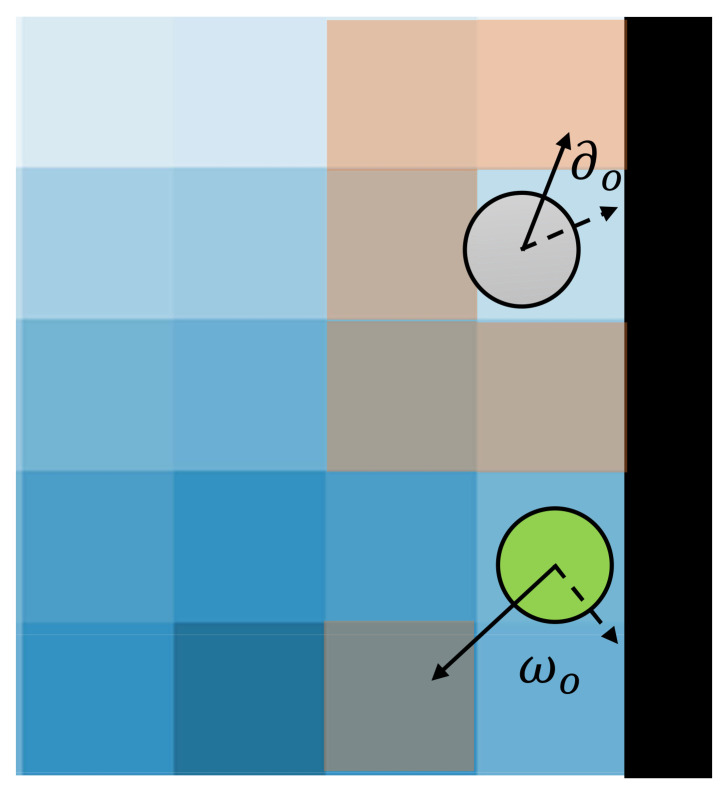
Evading behavior.

Evading behavior. The evading behavior refers to the action of evacuees in avoiding obstacles (e.g., walls or other evacuees). That is, this behavior prevents agents from colliding or overlapping with other evacuees, especially when the population density is high. In the ECBM, we define the exit area as a semi-circle with a radius around the exit. If an agent is close to the exit area, it only avoids overlapping with the obstacles because it aims to escape from the exit quickly. On the contrary, if an agent is far from the exit area, it tends to deflect as soon as it sees the obstacles. Figure 3 gives an example of the evading behavior. When facing the obstacles, non-guides (illustrated in the gray circle) deflect a stochastic angle $\partial_{o}$ within a maximum angle $\Phi_{o}$ toward one of the available neighboring grids to avoid the collision. However, guides (illustrated in the green circle) can choose the direction with a lower escape field value in the visible area instead of turning randomly.

**Figure 4:**
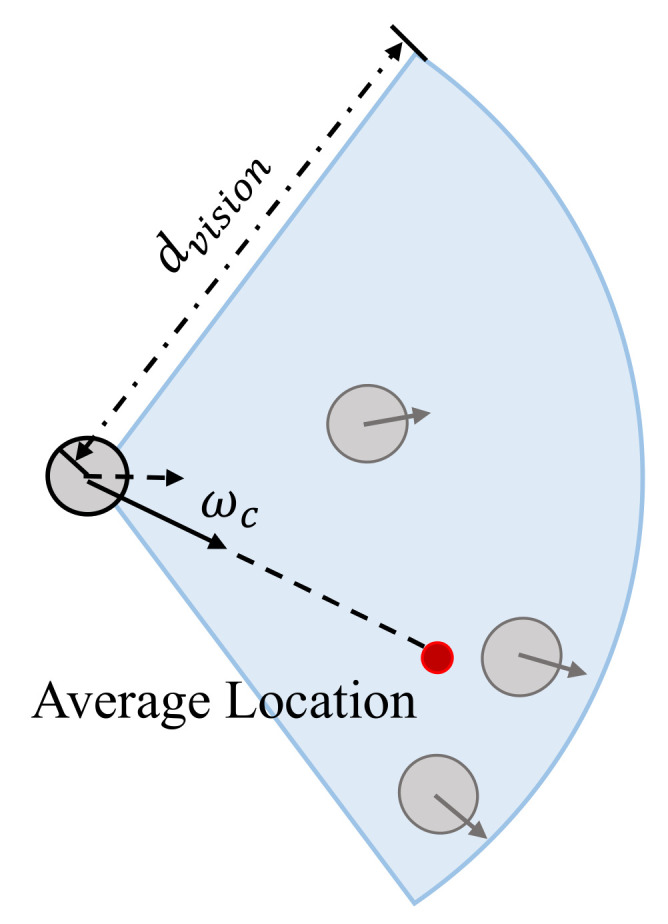
Cohering behavior.

Cohering behavior. Evacuees also tend to gather with the surrounding neighbors during an evacuation. In particular, the cohering behavior is often highlighted in studies on the herding behavior of evacuating crowds. The aggregation criteria in the Boids theory can describe this phenomenon. Specifically, the Boids theory [38] describes the self-organized phenomenon of bird flocks through the three basic rules of separation, alignment, and aggregation. The classical Boids model and its variants are widely used in flock simulations [43,44]. Similarly, agents in the ECBM turn toward the average position of nearby individuals (as shown in Figure 4). The parameter $\Phi_{c}$ is the peak of the steering angle $w_{c}$. When $\Phi_{c}$’s value is larger, the phenomenon of evacuating together is more obvious.

**Figure 5:**
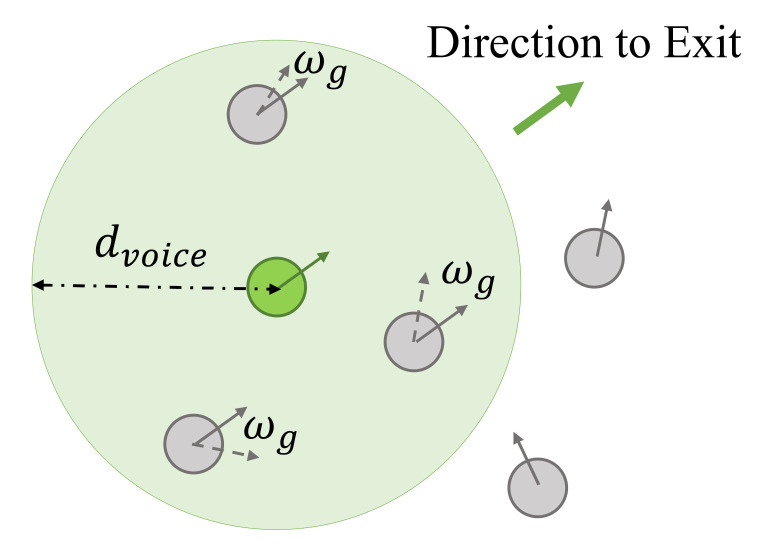
Guiding behavior.

Guiding behavior. If there are some people familiar with the architectural structure of the scene, they can directly move toward the exit instead of the lowest escape field. The guides always set the moving target of the escaping behavior as the exit direction rather than the direction with the lowest escape field in the vision field adopted by the non-guides. Because they know the location of the exits clearly and spread this information to the surrounding crowd, nearby agents within the voice scope can remain consistent with the guide. If there are non-guides within the sound range of the guide, the non-guides will follow the guide to move. Other non-guides not within the voice range of guides will not be guided and will show cohering behavior due to herd mentality. In the ECBM, we assume that guides in the crowd do not have a distinctive mark for visual recognition, but they can lead others by turning their head to expand their voice range. Thus, the influence range of guides is a circular area with a radius of sound propagation distance rather than the agent’s fan-shaped sound domain. Figure 5 shows an example of this behavior. The limit of angle $w_{g}$ is $\Phi_{g}$. The ECBM can simulate different lead–follow abilities of agents during an evacuation by adjusting the angle threshold.

**Figure 6:**
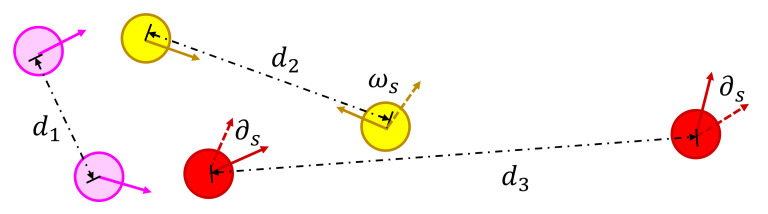
Illustration of the seeking behavior.

Seeking behavior. The seeking behavior is the particular actions of relatives in pairs. When the distance between two relatives exceeds a threshold, the agents seek their relatives instead of escaping toward the exit. Specifically, we consider that people intend to look for each other by sound, especially when sight is limited. We denote the sound propagation radius as $d_{\mathrm{voice}}$. Figure 6 gives an illustration of the seeking behavior. When the distance is less than the comfort range $d_{\mathrm{ease}}$ (e.g., the pink-colored agents), they do not seek each other. Therefore, their behavior rules are the same as those of independent agents. In contrast, when the distance is no less than $d_{\mathrm{ease}}$, the agents will detect whether their relatives are within their voice range or not. If so, the agents turn toward their partners as much as they can (e.g., the yellow-colored agents). Otherwise, the agents will turn randomly and keep seeking (e.g., the red-colored agents). The maximum of stochastic seeking angle $\partial_{s}$ is $\Phi_{s}$.

### 3.2. ECBM Algorithm

**Figure 7:**
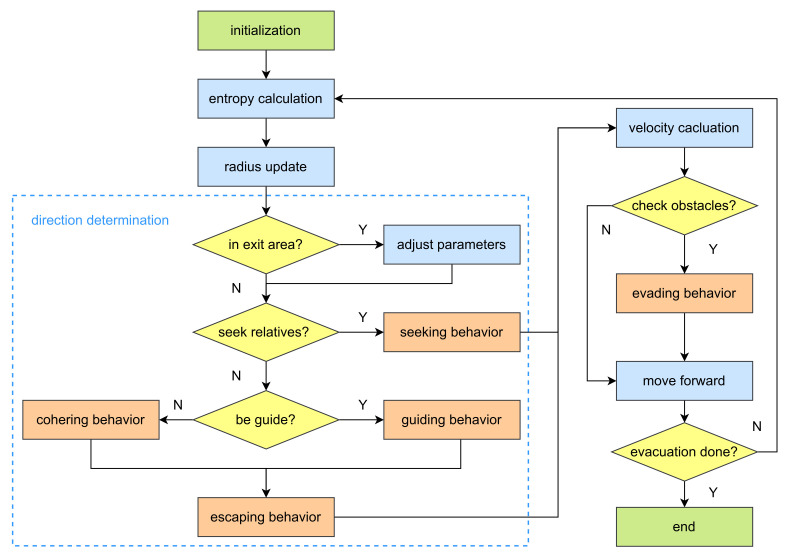
Algorithm flow of ECBM.

Based on the above five behavior modules, we introduce the proposed ECBM algorithm flow, as shown in Figure 7. After initialization, the agents iteratively execute the following steps until all the agents leave the exit.

**Figure 8:**
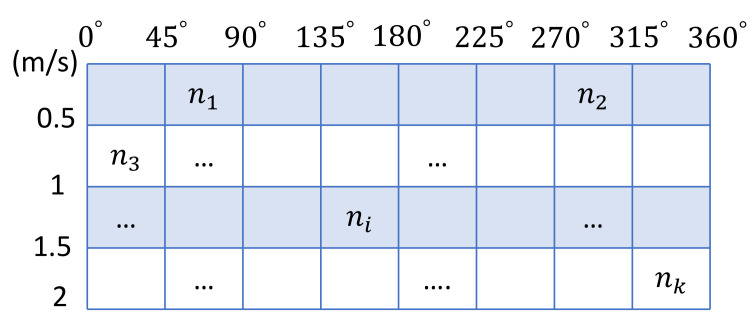
The velocity distribution of visible agents.

Entropy calculation. We first calculate the Boltzmann entropy for each agent given its visible range. Let *N* be the number of individuals in the *m*-th agent’s visible range. Given the magnitude and direction of velocity [45], we divide the space into a velocity distribution space with a 4 × 8 grid (as illustrated in Figure 8).

According to the definition, the Boltzmann entropy of a system reflects the degree of chaos of the system, and its calculation formula is: $BE=k_{B}*\ln W$, where $k_{B}$ is a constant and *W* represents the number of microscopic states of the system. For the evacuees, their micro-states are obtained by counting the probability distribution of pedestrian speed. Thus, we can calculate the velocity distribution of the *N* agents by $W=C_{N}^{n_{1}}*C_{N-n_{1}}^{n_{2}}*\cdots *C_{N-n_{1}-\cdots -n_{k-1}}^{n_{k}}$, where $\left\{\sum_{i=1}^{k}n_{i}=N\right\}$, $n_{i}$ is the number of agents in the *i*-th grid cell, and *k* is the number of cells with non-zero values. Then, at time *t*, the degree of chaos in the perspective of the *m*-th agent is:

(1) $$E_{m}\left(t\right)=k_{B}\cdot \ln\left(\Pi_{i=1}^{k}C_{N-\sum_{j=1}^{i-1}n_{j}}^{n_{i}}\right).$$

The larger the entropy value, the higher the disorder level of the crowd. We explain how to utilize this entropy value to reflect crowd chaos and update the agent’s velocity below.

Radius update. Then, we update the vision and voice radius based on the calculated entropy. The rationale is that the chaotic environment can affect humans’ sound range and visual field, which fluctuate at every time step. Typically, the radius is restricted if the crowd is more chaotic. Let S and L denote the sound and light propagation distances in the room, respectively. Thus, the parameters $d_{\mathrm{voice}}$ and $d_{\mathrm{vision}}$ are updated as follows.

(2) $$\begin{array}{cc} d_{\mathrm{voice}}\left(t\right) & =S/[1+E_{m}\left(t\right)], \end{array}$$

(3) $$\begin{array}{cc} d_{\mathrm{vision}}\left(t\right) & =L/[1+E_{m}\left(t\right)]. \end{array}$$

Direction determination. Next, each agent determines the evacuation direction for the next time step. Specifically, each agent first checks whether it is in the exit area and adjusts the parameters (e.g., destination and turning angles). Considering the influence of the exit visibility and high-density congestion on individual evacuation behavior, this paper sets parameter adjustments to make pedestrian evacuation behavior more realistic. When the distance of the pedestrian exit is less than 5 m, it means entering the visible area of the exit. In this exit area, the moving target of all pedestrians’ escaping behaviors is the exit center. Because of the sense of security brought by the visibility of the exit and the pressure caused by the high density of arched congestion, the pedestrians’ herd mentality is weakened. Therefore, the value of the pedestrians’ aggregation angle is halved in the exit area. To avoid overlapping between pedestrians and obstacles, pedestrians in the exit area still have evading behavior when moving and may change the direction of movement to stay away from the nearest obstacle. Then, if the agent has a relative, it will seek the relative, where the seeking behavior determines the direction (see Section 3.1). If the agent does not have a relative or its relative is within the comfort range $d_{\mathrm{ease}}$, we further check whether the agent is familiar with the evacuation environment. If so, the guiding behavior will be triggered. Otherwise, the agent will perform the cohering behavior, i.e., gather with the nearby individuals (moving toward the direction of the average position). Finally, we calculate the direction of the escaping behavior (i.e., the direction to the exit) for each agent and aggregate it to obtain the final direction.

**Figure 9:**
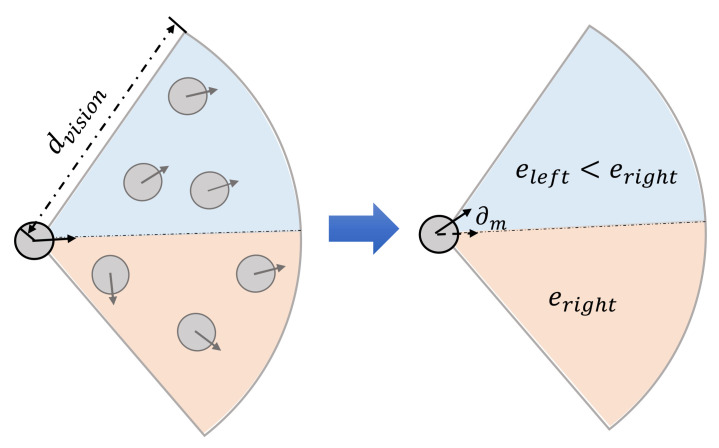
Illustration of direction modification based on entropy.

Velocity calculation and adjustment. After determining the direction through behavior rules, we now calculate each agent’s velocity. There are two designs. First, we observe that evacuees tend to show unilateral preference when moving. However, the unilateral preference considered in existing studies is mainly a right-sided preference [46,47] and experiments have shown that a right-sided preference may lead to an increase in the crowd congestion density and a decrease in moving speed [48]. Thus, considering the evacuees’ perceptions of the chaos degree of the visible agents, we let each agent move in a direction that avoids local chaos in the visual field. Figure 9 illustrates this design, where the agent deflects to the more orderly side (according to the calculated Boltzmann entropy). The range of the drift angle $\partial_{m}$ is $\left[0,\Phi_{m}\right]$, where $\Phi_{m}$ is the maximum modification angle.

Second, we also take the entropy into the calculation of each agent’s next step velocity. Let V(t) and $V_{\mathrm{aver}}\left(t\right)$ be the current velocity of an agent and the average velocity of its neighbors. Specifically, we update the velocity of the agent as follows based on the exponential smoothing method.

(4) $$V\left(t+1\right)=\left[1-E_{m}\left(t\right)\right]*V\left(t\right)+E_{m}\left(t\right)*V_{\mathrm{aver}}\left(t\right),$$

which means that the individual speed is influenced by an entropy (i.e., crowd chaos degree) and the average speed of neighboring agents. Note that the velocity calculation is more consistent with the real-world scenario: when $E_{m}\left(t\right)$ is larger, the environment is more chaotic and individuals will tend to follow the crowd to move; when $E_{m}\left(t\right)$ is smaller, the environment is more orderly and individuals can move according to the previous decision.

Evading and moving. Before moving to the next position, the last step is to ensure that there are no collisions with the obstacles. Specifically, each agent checks whether an obstacle (including other agents and walls) is nearby and deflects with a random angle, as discussed in Section 3.1. As a consequence, each agent moves to the designated position and continues the next iteration until it leaves the exit.

## 4. Simulation and Analysis

Now, we evaluate the ECBM evacuation model using simulations. We implemented the evacuation model and simulated the evacuation process based on NetLogo 6.2.2 https://ccl.northwestern.edu/netlogo/ (accessed on 1 July 2022), which is a multi-agent programmable modeling environment. We conducted the experiments on a machine that runs Windows 10 with an Intel (R) Core (TM) i5-8265U CPU @ 1.60 GHz and 8.00 GB of RAM. Table 1 gives the default experimental parameters. The evacuation scene was a $31\times 31\;m^{2}$ room. There was an exit with a width of 3m. The default total agent number in the simulation was 200, and the agent’s radius was 0.4 m. The individual had a visual field with a radius of 5 m [41]. Because the hearing radius was not blocked by other pedestrians unlike the visual radius, it was slightly higher than the visual radius and set to 8 m. The comfortable distance and individual speed limit followed the settings used in previous research [49], with values of 2 m and 2 m/s, respectively.

In the following, we first investigate the effect of entropy on the evacuation process in Section 4.1. Then, we evaluate the effects of the seeking behavior and guiding behavior in Section 4.2 and Section 4.3, respectively. Finally, we explore the impact of the behaviors’ parameter values on an evacuation in Section 4.4.

### 4.1. Effect of Entropy on Evacuation

**Figure 10:**
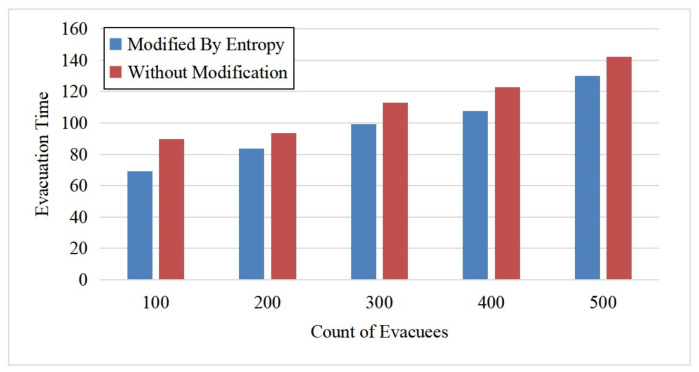
Comparison of evacuation time for the proposed entropy-based velocity adjustment method and the method without entropy adjustment.

**Figure 11:**
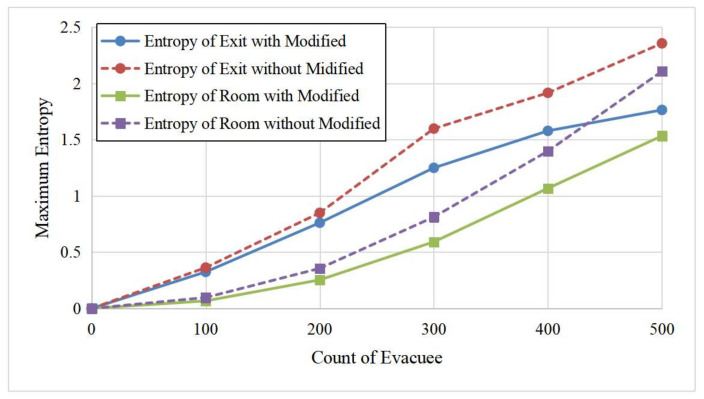
Comparison of crowd entropy for the whole room and exit area for the proposed entropy-based velocity adjustment method and the method without entropy adjustment.

In this set of experiments, we set all the evacuees in the ECBM as independent agents and varied the number of evacuees (100, 200, 300, 400, and 500). Figure 10 shows a comparison of the evacuation time for the evacuation with a modified velocity by entropy (i.e., Equation (4)) and the evacuation without modification. There are two main observations. First, the evacuation time increased as the number of evacuees increased, which is reasonable because it should take a longer time to evacuate more people. Second, the evacuation time was shorter when applying our entropy-based velocity calculation and adjustment method. The reason is that the agent tended to avoid the chaotic area during the evacuation, making the overall evacuation more efficient and demonstrating our evacuation model’s effectiveness. Figure 11 illustrates the chaos level of the room and the exit area for the above two methods. We can see that the maximum Boltzmann entropy of the exit area with our proposed velocity calculation method was smaller than the method without modifications. Moreover, the more evacuees in the room, the better the effect of the entropy-based velocity method. This phenomenon also shows that if people are able to avoid chaotic areas during the escape, the evacuation will be more efficient and orderly.

### 4.2. Effect of Seeking Behavior on Evacuation

**Figure 12:**
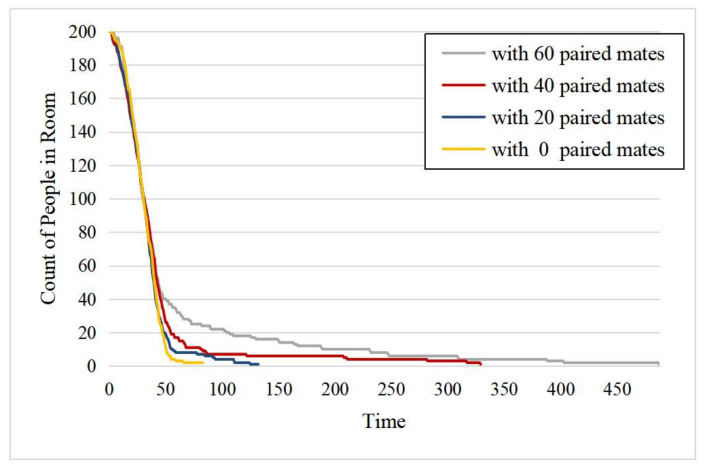
The number of remaining evacuees in the room.

In this set of experiments, we considered the paired small social groups (also known as mates) in the evacuees. Figure 12 shows the remaining number of evacuees in the room with respect to the evacuation timestamps for the different numbers of small social groups (i.e., 0, 20, 40, and 60). We observed that as the number of paired mates increased, the time for the evacuation process was longer, indicating that the seeking behavior directly increased the evacuation time. This is because more evacuees looked for each other instead of following the escape routes. In addition, the falling speed of the remaining crowd slowed down from the 50th second. However, when more paired mates were in the crowd, more people left at the 50th second, and thus, the longer they took to complete the evacuation.

**Figure 13:**
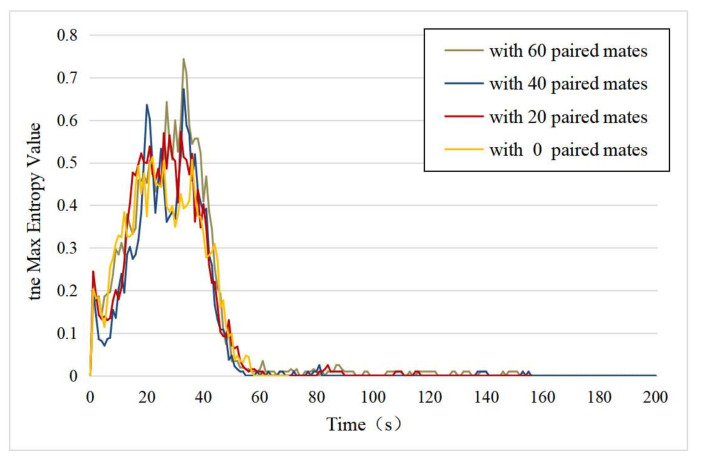
The maximum entropy of evacuees.

We also reported the maximum entropy of the evacuees during the first 200 s, as depicted in Figure 13. From the 20th to the 40th seconds, we observed that the crowd was the most chaotic and the peak value of the entropy increased with more pairs of small social groups. This shows that small social groups can aggravate the disorder level of the crowd. Moreover, the higher the proportion of small social groups in the crowd, the greater the fluctuation in the range of entropy, indicating that small social groups also increase the uncertainty of the crowd chaos degree.

Based on the above simulation results, we confirm that the seeking behavior of social groups is a critical factor affecting evacuation efficiency. In some special scenarios, such as children’s indoor amusement parks, the behavior of searching for relatives makes the behavior characteristics of the crowd different to general scenes. We highlight the necessity of designing more suitable emergency plans for these scenarios.

### 4.3. Effect of Guiding Behavior on Evacuation

**Figure 14:**
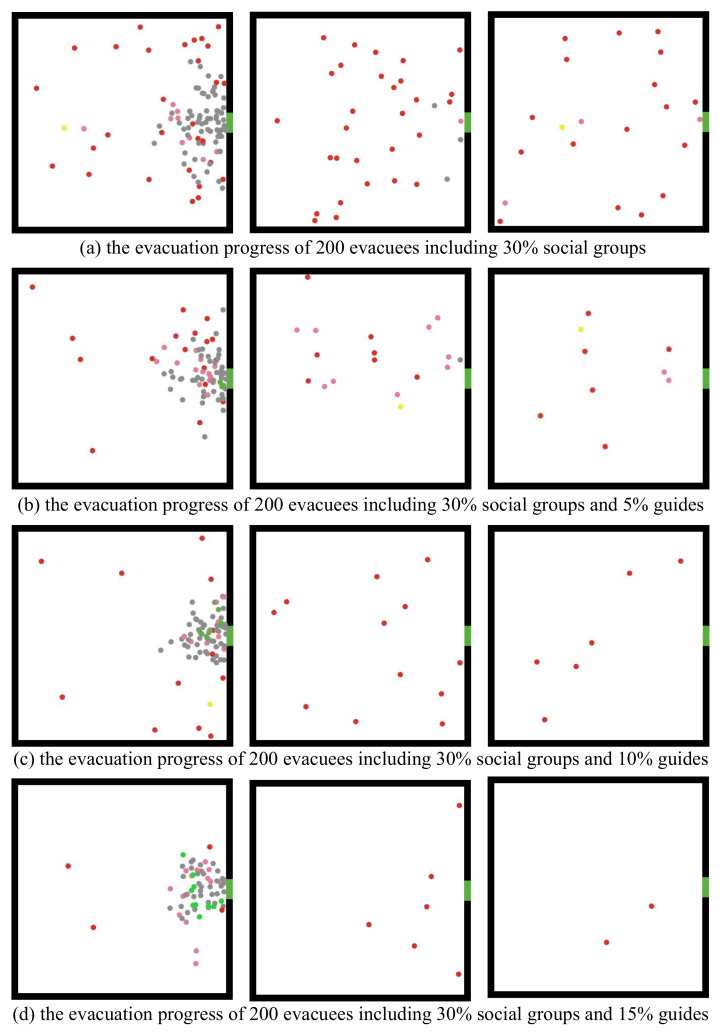
The early, middle, and late stages (from left to right) of evacuation for 200 people with 60 paired small social groups and 0, 10, 20, and 30 (from top to bottom), respectively, guides.

In this subsection, we consider a scenario where some people in the crowd are familiar with the evacuation scene such as staff and residents; they can quickly determine the direction of the exits and convey the message to others around them. It is worth noting that, unlike guidance signs or professional rescuers, the guides in this paper keep moving toward the exit while guiding others rather than stopping or retreating. Figure 14 illustrates the evacuation process concerning different numbers of guides (i.e., 0, 10, 20, and 30), where gray agents represent individuals; green agents represent guides; and pink, yellow, and red agents represent the members of the social groups in the early, middle, and late stages. The simulation showed that the existence of guides could effectively reduce the number of agents searching for relatives. Specifically, we observed that guides could lead the crowd to gather at the exits faster during the early stage of an evacuation, and the number of people seeking relatives in the room also decreased significantly. Furthermore, we observed that the model reproduced the typical exit arch congestion phenomenon of crowd evacuation, demonstrating the effectiveness of our evacuation model. This qualitative verification, that is, the reproduction of the phenomenon of the self-organization of the evacuation population [50], is one of the most commonly used verification methods in micro evacuation models. In our future work, we plan to further study the quantitative verification of the model, analyze the fundamental diagram [51,52] of the pedestrian flow in the corridors, and compare the errors in the model simulation data and the real evacuation drill data [53].

**Figure 15:**
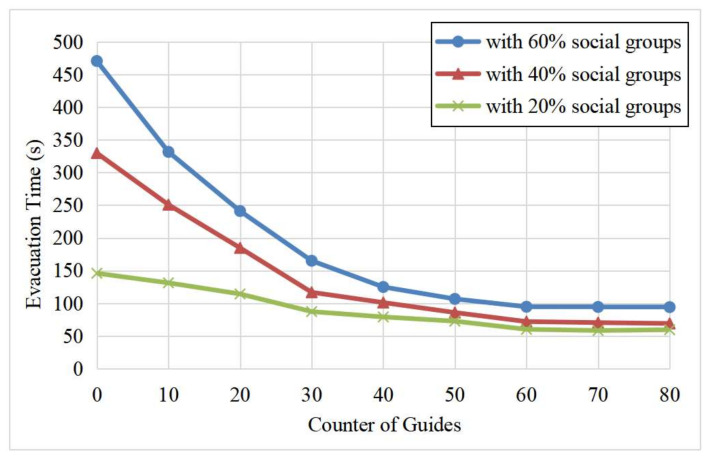
Average egress times under different guide proportions.

Figure 15 shows that the improvement in the evacuation time tended to be saturated with the increase in the number of guides. The evacuation time could be reduced when there were guides in the crowd. For example, when the number of guides was less than 40, the more guides in the crowd, the higher the evacuation efficiency. However, when the number of guides exceeded 60, the evacuation time was almost unchanged. That is, the effect was saturated because the guides already exceeded 30% of the population (given 200 evacuees in the simulation).

### 4.4. Effect of Parameter Values on Evacuation

**Figure 16:**
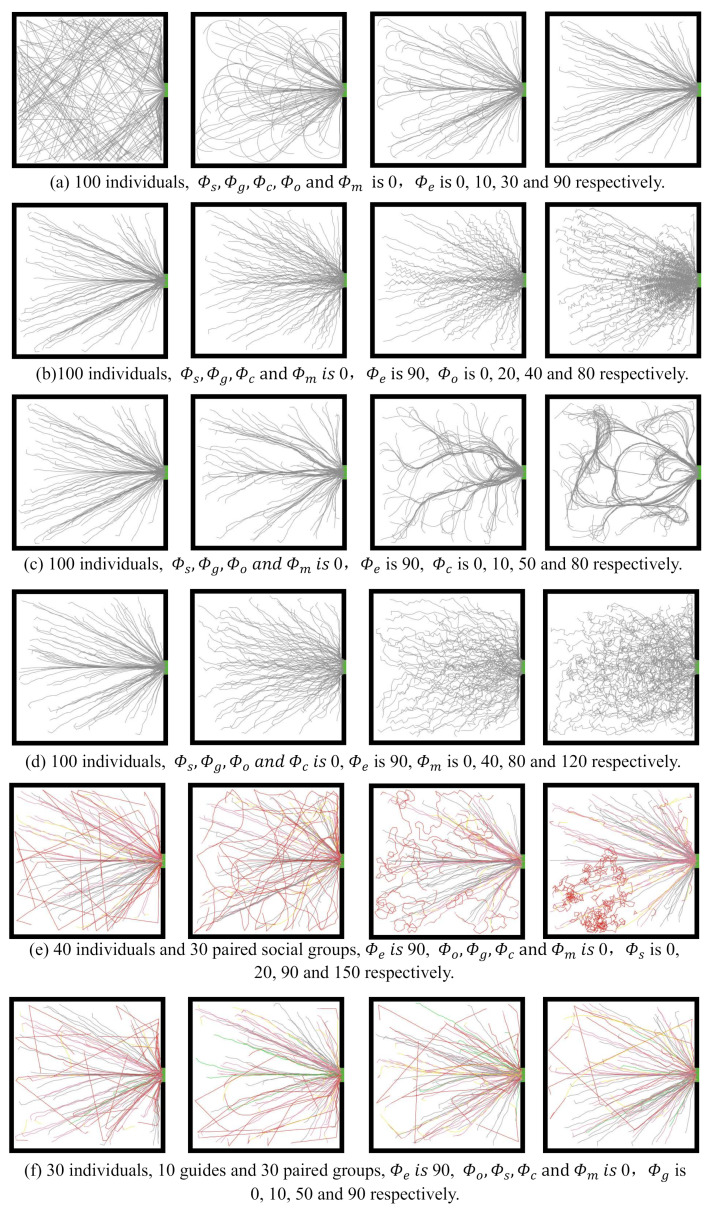
Evaluation of different parameter values of maximum angles in ECBM.

We further analyzed the influence of the maximum angle parameters in the ECBM on individual evacuation behavior. Figure 16 shows the crowd evacuation trajectories under different parameter conditions. Unless noted otherwise, the maximum angle for the escaping behavior $\Phi_{e}=90$ and the other parameter values are 0 by default.

Figure 16a–d shows the evacuation trajectories of 100 individuals. Specifically, Figure 16a illustrates the crowd evacuation trajectories by varying $\Phi_{e}\in \left\{0,10,30,90\right\}$, respectively, whereas the other parameters are 0. When $\Phi_{e}$ = 10, based on the escape field in the visual field, the evacuee’s turning and moving processes from the initial speed direction to the exit direction were slower and formed a circular arc trajectory, which was not reasonable. In contrast, this phenomenon disappeared when $\Phi_{e}\geq 90$. Therefore, the value range of this parameter is [90, 360].

Figure 16b shows the crowd evacuation trajectories when $\Phi_{o}\in \left\{0,20,40,80\right\}$, respectively. As the value of $\Phi_{o}$ increased, the evacuation trajectory showed a zigzag pattern. When $\Phi_{o}\geq 40$, a large zigzag trajectory range appeared, which is not in line with the actual movement of the crowd. Therefore, the value range is [0, 40]. In addition, with the increase in $\Phi_{o}$, it was observed that the range of the arched areas with dense moving tracks near the exit increased. This is because the larger the obstacle avoidance angle, the greater the tendency of pedestrians to maintain distance between each other, leading to the distribution of people near the exit being more dispersed.

Figure 16c shows the crowd evacuation trajectory given $\Phi_{c}\in \left\{0,10,50,80\right\}$, respectively. When $\Phi_{c}=10$, we observed a relatively obvious crowd cohering phenomenon. In addition, as the value of $\Phi_{c}$ increased, the degree of cohering intensified. When $\Phi_{c}=50$, a detour occurred due to excessive cohering, and the detour became more severe as $\Phi_{c}$ continued to increase. Therefore, the value range of $\Phi_{c}$ is [10, 50], which is more consistent with an actual evacuation situation.

Figure 16d shows the crowd evacuation trajectory given $\Phi_{m}\in \left\{0,30,50,80\right\}$, respectively. As $\Phi_{m}$ increased, the evacuee trajectory became more scattered and zigzagging, and the space utilization on both sides of the exit became higher. However, when $\Phi_{m}=120$, the trajectory was abnormally curly, indicating that the velocity adjustment was excessive and the individual turned too frequently. Since the velocity adjustment is related to the individual’s visual field (the default visual angle was 170 in this paper), it is reasonable that the one-sided preference deflection angle was less than 85 degrees. The parameter $\Phi_{m}$ reflects the preference intensity of individuals to avoid local chaos and its value range is [0, 85].

Figure 16e shows the evacuation trajectories of a heterogeneous crowd composed of 40 individuals and 30 paired small groups when $\Phi_{s}\in \left\{0,20,90,150\right\}$, respectively. We observed that the direction of the seeking trajectories changes less when $\Phi_{s}\leq 20$. Meanwhile, when $\Phi_{s}=90$, many arc-shaped seeking tracks could be seen. The seeking trajectories may be densely folded when $\Phi_{s}\geq 150$. $\Phi_{s}$ represents the maximum range in which small social groups can move in search of unseen companions. The larger the $\Phi_{s}$ value, the higher the uncertainty of the distribution range and duration of the tracing trajectory of the small group. The suggested value range of this parameter is [30, 200], considering the turning limit of evacuees within one second.

Figure 16f shows the evacuation trajectories of a heterogeneous crowd composed of 40 individuals, 10 guides, and 30 paired small groups when $\Phi_{g}\in \left\{0,10,50,90\right\}$, respectively. When $\Phi_{g}=0$, the seeking trace was straight because $\Phi_{s}$ was 0. When $\Phi_{g}=10$, some evacuees seeking relatives were guided, thus forming many arc-shaped steering trajectories. As $\Phi_{g}$ increased to 50, the arc trajectory disappeared. Through simulations, we observed that the steering angle caused by the guiding behavior was usually less than 90. Therefore, the value range of $\Phi_{g}$ is [50, 90]. Adjusting this parameter can change the guiding effect of the guides.

## 5. Conclusions

In this paper, we proposed an entropy-based combined behavior model ECBM for crowd evacuation. The proposed model incorporates crowd chaos into the evacuation process, which is measured by the Boltzmann entropy. Meanwhile, we simulate the evacuee’s evacuation through a combination of five behavior rules. Furthermore, we devise a velocity adjustment method to calculate the agent’s velocity based on the crowd chaos level, which is more consistent with realistic evacuation scenarios. Finally, we conduct extensive simulation experiments, demonstrating the effectiveness of the proposed evacuation model and providing practical information for the design of evacuation strategies.

## Figures and Tables

**Table 1:** Experimental parameters.

Notation	Description	Default Value
*S*	sound propagation distance in evacuation scenario	8 m
*L*	light propagation distance in evacuation scenario	5 m
$d_{\mathrm{ease}}$	the distance to determine whether seeking relatives	2 m
$\Phi_{m}$	the maximum angle of entropy modification	10°
$\Phi_{s}$	the maximum angle of seeking relatives	150°
$\Phi_{e}$	the maximum angle based on the escaping field	90°
$\Phi_{g}$	the maximum deflection angle when being guided	180°
$\Phi_{c}$	the maximum angle toward the nearby groups	20°
$\Phi_{o}$	the maximal turning angle to avoid obstacles	20°
$v_{\max}$	the maximal speed of evacuees	2 m/s

## Data Availability

Not applicable.
